# Eccentric Exercise Activates Novel Transcriptional Regulation of Hypertrophic Signaling Pathways Not Affected by Hormone Changes

**DOI:** 10.1371/journal.pone.0010695

**Published:** 2010-05-18

**Authors:** Lauren G. MacNeil, Simon Melov, Alan E. Hubbard, Steven K. Baker, Mark A. Tarnopolsky

**Affiliations:** 1 Department of Kinesiology, McMaster University, Hamilton, Ontario, Canada; 2 Buck Institute for Age Research, Novato, California, United States of America; 3 School of Public Health, University of California, Berkeley, California, United States of America; 4 Department of Medicine, McMaster University, Hamilton, Ontario, Canada; 5 Department of Pediatrics, McMaster University, Hamilton, Ontario, Canada; Universidad Europea de Madrid, Spain

## Abstract

Unaccustomed eccentric exercise damages skeletal muscle tissue, activating mechanisms of recovery and remodeling that may be influenced by the female sex hormone 17β-estradiol (E2). Using high density oligonucleotide based microarrays, we screened for differences in mRNA expression caused by E2 and eccentric exercise. After random assignment to 8 days of either placebo (CON) or E2 (EXP), eighteen men performed 150 single-leg eccentric contractions. Muscle biopsies were collected at baseline (BL), following supplementation (PS), +3 hours (3H) and +48 hours (48H) after exercise. Serum E2 concentrations increased significantly with supplementation (P<0.001) but did not affect microarray results. Exercise led to early transcriptional changes in striated muscle activator of Rho signaling (STARS), Rho family GTPase 3 (RND3), mitogen activated protein kinase (MAPK) regulation and the downstream transcription factor FOS. Targeted RT-PCR analysis identified concurrent induction of negative regulators of calcineurin signaling RCAN (P<0.001) and HMOX1 (P = 0.009). Protein contents were elevated for RND3 at 3H (P = 0.02) and FOS at 48H (P<0.05). These findings indicate that early RhoA and NFAT signaling and regulation are altered following exercise for muscle remodeling and repair, but are not affected by E2.

## Introduction

Myofibres have the capacity to be remodeled to best meet their functional and metabolic demands. Changes in physical activity can initiate a remodeling process toward increased (hypertrophy) or decreased (atrophy) muscle mass [1], [2], [3]. Through protein signaling pathways, integration of chemical, mechanical and bioenergetic signals change the genetic expression patterns for cell size, function and metabolic processes [4]. Physical activities that incorporate unaccustomed eccentric contractions are typically associated with high levels of muscle damage, inflammation and delayed onset muscle soreness (DOMS) [5], [6], [7]. Eccentric contractions, identified by a lengthening while under tension, create an insult to myofibres which may be characterized by: damage to the sarcoplasmic reticulum [5], t-tubules [8] and structural proteins [6], [9], [10], the presence of muscle protein in blood [11], [12], Z-line streaming [13], [14], soreness and fatigue [7], [8].

Murine and rodent research has often indicated an attenuation of exercise induced membrane damage [15], [16], [17], [18], [19], structural proteins [20], and inflammation [21], [22], [23], [24] along with enhanced satellite cell activation [21], [25], [26] with exposure to the sex hormone 17β-estradiol (E2). This reduced muscle damage and improved recovery may result from potential antioxidant, membrane stabilizing, or gene regulation properties of E2 [27], [28], [29], [30], [31]. As the most abundant estrogen, E2 exerts estrogenic properties that affect the differentiation, growth and function of reproductive, skeletal, neural and muscular tissues [31], [32]. However, human studies do not consistently support the effectiveness of E2 to attenuate exercise-induced muscle damage, mostly reporting similar values for CK efflux, inflammation and loss of muscle function when comparing men and women [6], [33].

Recent microarray analyses have uncovered novel transcriptional programs that coordinate the regeneration and repair of damaged muscle following eccentric exercise [10], [34], [35]. These studies have indentified clusters of genes representing important mechanisms for recovery and adaptation that include the regulation of inflammation [10], [35], growth [10], [35], stress response proteins [10], [34] and membrane biosynthesis [34]. Similar analyses have also described sex differences in the expression levels of genes involved in metabolism and growth inhibition that may result from variations in body composition and hormone content [36], [37], [38]. Specifically, women express a higher abundance of mRNA for several genes involved in fat metabolism that include trifunctional protein β and lipoprotein lipase [36], and greater expression of the negative regulators of the anabolism growth factor receptor-bound 10 and activin A receptor IIB [37].

The effect of E2 administration on the transcriptome expression profile of skeletal muscle following a single bout of intense eccentric exercise has not yet been evaluated. In this study we used microarray analysis to identify how global mRNA abundance is altered by E2 supplementation in men after 150 eccentric contractions. We hypothesized that the anti-oxidant and membrane stabilizing properties of E2 would attenuate the amount of muscle damage experienced, thereby modifying the expression of mRNA species involved in membrane homeostasis, growth and stress management. Furthermore, we hypothesized that the use of gene array analysis would allow us to detect novel genes relevant to the hypertrophic growth signaling stimulated by intense exercise.

## Materials and Methods

### Ethics statement

All participants were given an information sheet describing all of the testing procedures before providing written consent to participate. The study conformed to the standards outlined in the *Declaration of Helsinki* and was given approval by the Research Ethics Board of McMaster University (05–438).

### Subjects and anthropometrics

Eighteen young healthy men volunteered as participants in this study. All subjects were pre-screened to ensure that they were healthy, fit and had not regularly participated in resistance exercise in the preceding 6 months. Body composition was measured using dual energy x-ray absorptiometry (DEXA) scans (GE Lunar Prodigy, Fairfield, CT). Thigh muscle cross-sectional area was calculated using anthropomorphic measurements of mid-thigh circumference and skinfold thickness [39] to control for potential differences in total work completed. The subject demographics were (mean ± SD): age, 21±2 y; height, 181±5 cm; weight, 76.9±12.8 kg.

### Supplementation protocol

Subjects were assigned in a randomized, double-blind manner to either a control (CON, N = 9) or experimental (EXP, N = 9) group. CON subjects consumed 400 mg glucose polymer (Polycose; Abbott Laboratories, Ross Division, St. Laurent, Quebec, Canada) for 10 days. EXP subjects consumed ∼300 mg glucose with 1 mg E2 (Estrace; Shire BioChem, Inc., St. Laurent, Quebec, Canada) for 2 days followed by 2 mg E2 for 8 days. We have previously used this protocol to increase serum E2 concentrations to levels seen during the luteal phase of the menstrual cycle [40]. Glucose and E2 tablets were concealed in gelatin capsules. On the morning of the ninth day, subjects reported to the laboratory and performed the exercise protocol. Supplementation continued until the day of the final biopsy and blood collection to maintain serum E2 concentrations throughout the collection protocol. Subjects in both groups were instructed to take one pill at the same time each day and return any unused pills. All subjects reported 100% compliance.

### Exercise protocol and tissue collection

Muscle damage was induced with a previously developed eccentric exercise protocol [41]. Approximately 2 weeks before the exercise protocol, subjects were given a familiarization session with a Biodex isokinetic dynamometer (System 3, Biodex Medical Systems Inc., Ronkonkoma, NY). On the testing day, following a short warm-up (10 min of light cycling), subjects were seated in the dynamometer with their right leg strapped to a lever arm. The lever arm was programmed to extend their leg to 150° of flexion (where 180° is full extension) at a moderate speed (30°/s), then flex their leg to 90° of flexion at a faster speed (120°/s). Subjects did not have to contract maximally during the extension phase. During the flexion phase, subjects were instructed to attempt to maximally resist flexion of the knee (i.e. voluntary ‘maximal’ contraction) against the descending lever arm throughout the entire range of motion. The complete test consisted of 15 sets of 10 repetitions, each set separated by 1 minute of rest.

Prior to each tissue collection, subjects abstained from any other form of physical exertion (within 72 h), avoided alcohol (within 48 h), ate their habitual diet (within 48 h), and abstained from caffeine (within 12 h). Each subject consumed a 350 Kcal defined formula diet (57% carbohydrates, 15% protein and 28% fat) two hours before each muscle biopsy and did not eat again until after the final biopsy of each session was taken. These nutritional and activity controls were taken to ensure that the muscle damage would be the only variable to differentially affect the outcomes between biopsies [42].

Muscle biopsies were taken from the vastus lateralis of the control (left) leg during the familiarization session (baseline, BL) and after 8 days of supplementation (post supplementation, PS) and the exercised (right) leg 3 hours (3H) and 48 hours (48H) after exercise, in anatomically distinct sites approximately 6 cm apart [43]. The post exercise collection times were chosen because they represent two distinct phases of recovery from muscle damage [6]. The muscle biopsies were quickly dissected of fat and connective tissue, sectioned into RNase-free cryovials (∼30 mg/piece), flash frozen with liquid nitrogen and stored at −86°C until analysis. Blood samples were drawn from the antecubital vein into heparinized tubes at the same collection times, placed on ice, centrifuged at 1750 g at 4°C for 10 min and stored at −20°C future analyses.

### Blood hormone and enzyme concentrations

Serum E2 (Fertigenix-E2-EASIA, Biosource Europe S.A, Nivelles, Belgium) and testosterone (Fertigenix-TESTO-EASIA, Biosource Europe S.A, Nivelles, Belgium) concentrations were measured by enzyme amplified-sensitivity immunosorbent assays (EASIA) according to manufacturer's specifications using BL and PS blood collections. Serum lactate dehydrogenase (LDH) activities were measured in BL, 3H and 48H blood collections with a colourimetric LDH quantification assay (K726-500, Biovision Research Products, Mountain View, CA) according to manufacturer's specifications. All hormone and enzyme measurements were done in duplicate.

### RNA extraction

The total RNA was extracted from the frozen skeletal muscle biopsy as described previously in detail by our group [43]. Briefly, ∼30 mg of skeletal muscle was homogenized on ice in 2 mL of Trizol Reagent (Life Technologies, Cat. No. 15596, Gaithersburg, MD). The homogenate was incubated for 10 min at room temperature, followed by phase separation using 200 µL of chloroform and precipitation of the total RNA from the aqueous phase using 500 µL of isopropyl alcohol. The RNA pellet was then washed three times in 75% ethanol and re-suspended in 15 µL DEPC-treated water, aliquoted, and stored at −86°C. The concentration and purity of the RNA was determined using a UV spectrophotometer (Shimadzu UV-1201; Mandel Scientific, Guelph, Ontario) at the absorbance of 260/280 nm. Measurements were done in duplicate and had an average coefficient of variation (CV) of <10%. The average purity (OD_260_/OD_280_) of the samples was 1.7 before DNase treatment. RNA integrity was assessed in a randomly chosen subset of samples using agarose gel electrophoresis, and the OD ratio of 28S to 18S rRNA was consistently greater than 1 for each sample.

### DNase treatment

Prior to microarray chipping and real time quantitative RT-PCR analysis, the isolated RNA samples were treated with DNA-free™ recombinant DNase I (Ambion Inc, Austin, TX) according to the manufacturer's instructions to remove any potential genomic DNA contamination.

### Microarray analysis

The resulting total RNA samples were further assessed for integrity prior to chipping using a Nanodrop Spectrophotometer and the Agilent Bioanalyzer Nano Chip System. Samples which passed this initial quality control assurance step were then amplified one round, using an Illumina TotalPrep Kit (Ambion) to generate cDNA then cRNA according to the manufacturer's instructions. This was again assessed for quality by using the Nanodrop and Bioanalyzer as described above. Labeled cRNA samples that passed this second round of quality control were then hybridized to Human Ref-8 BeadChips (Illumina) according to the manufacturer's instructions (approximately 23,000 genes), using equipment specified by the manufacturer (Illumina). Briefly, 850 ng biotin-labeled cRNA in 11.3 µl nuclease-free water was adjusted to 34 µl through the addition of 22.7 µl of 5∶3 HybE1 buffer/formamide. The sample was heated at 65°C for 5 min, allowed to cool to room temperature, and then immediately added to a single array of an 8-array Human Ref-8 BeadChip. Once all 8 samples were added to each BeadChip, it was sealed in a Hyb Cartridge and incubated for 16 h at 55°C with rotation in an Illumina hybridization oven (rotation setting 5). Following overnight hybridization, BeadChips were moved to a slide rack and serially washed using gentle rotation in glass staining dishes filled with a) 250 ml Illumina Wash Buffer×5 min, b) 250 ml 100% ethanol×10 min, c) 250 ml Illumina Wash Buffer×2 min. BeadChips were then blocked for 10 min in 4 ml Block E1 buffer (Illumina), followed by staining for 10 min in 1 µg/ml Streptavidin-Cy3 conjugate (GE Healthcare) in Block E1 buffer. Stained BeadChips were finally washed using gentle rotation in a glass staining dish filled with 250 ml Illumina Wash Buffer×5 min. BeadChips were dried by centrifugation at 280 *g* for 4 min and stored in a light-tight box until reading.

### Array reading

Processed arrays were read using a BeadStation array reader (Illumina) according to the manufacturer's instructions.

### Gene ontology analysis

In the lists of genes that were significantly differentially expressed with exercise in our study, we carried out gene ontology (GO) analysis to determine the relative enrichment of genes with common or related functionalities to gain insight into biological processes mediated by E2 or exercise. This was carried out using the web interface driven GoMiner tool using an FDR of 5% [44]. Genes were also referenced to their biological functions and canonical pathways with Ingenuity Pathway Analysis (IPA) software. This software identifies functions and pathways most significant to the data set in two ways: the number of differentially expressed genes included in a pathway or function and calculation of a *p*-value using a Fisher's Exact Test to determine the probability of the association of the data set.

### Real-time RT-PCR analysis

Changes in gene expression relative to baseline values were measured using real-time reverse transcription-polymerase chain reaction (RT-PCR). Regulator of calcineurin 1 (RCAN1) and capping protein (actin filament) muscle Z-line, alpha 1 (CAPZA1) were selected for analysis because of their roles in growth and sarcomerogenesis [34]. Hemeoxygenase 1 (HMOX1) was chosen for analysis because of its role in stress management [45]. The selected housekeeping gene was β2-microglobulin. Its constant expression following eccentric exercise has been shown in previous work [43], and was confirmed for the current study. The efficiencies of all primers were tested and determined to be greater than 98%. The primer and probe sequences for these genes can be found in Table 1.

**Table 1:** Primer and probe sequences for calcineurin regulation, actin dynamics and housekeeping genes.

Gene	Left Primer	Right Primer	Probe
RCAN1	gacaaggacatcacctttcagt	tcatttcctttcccagaaactc	caaacgagtcagaataacttcagcaaccc
HMOX1	tctccgatgggtccttacac	cctgcattcacatggcataa	ctaagccaactgtcgccaccagaaa
CAPZA1	ccttccaagcacttctggtact	gagggagaaggatgaatgtgt	atccaccaacacctaaagaggctatgc
β2M	ggctatccagcgtactccaa	gatgaaacccagacacatagca	tcaggtttactcaacgtcatccagcagag

RT-PCR was completed using a TaqMan® real-time method. The primers and a probe to each target gene were designed based on the cDNA sequence in GenBank (http://www.ncbi.nlm.nih.gov/sites/entrez/?db=gene) with primer 3 designer (http://frodo.wi.mit.edu/primer3-0.4.0/input.htm). All target gene probes were labeled with FAM at their 5′ ends and BHQ-1 at their 3′ ends. Duplex RT-PCR was performed on an iCycler real-time PCR system (Bio-Rad Laboratories, Hercules, CA) in the One-step TaqMan® RT-PCR Master Mix Reagents (Roche, Branchburg, New Jersey) according to the manufacture's instruction with target gene primers, target probe, housekeeping gene primers and housekeeping gene probe in the same reaction [46]. Determination of significant gene expression change was done as previously described [46]. The genes of interest were normalized to the housekeeping gene, β2-microglobulin by following the standard method. Briefly, C_T_ values of the housekeeping gene were subtracted from the C_T_ values of the gene of interest giving a δC_T_. This is equivalent to the log_2_ difference between endogenous control and target gene [47]. Values were then normalized to baseline, δδC_T_. All samples were run in triplicate, fluorescence emission was detected using FAM and Tamra filters, and C_T_ was automatically calculated.

### Western blotting

Muscle biopsy samples were homogenized and prepared for polyacrylamide gel electrophoresis using methods previously described [48]. Briefly, frozen skeletal muscle tissue samples (25–35 mg) were hand homogenized in 25 µl of phosphate buffer (50 mM Kpi, 5 mM EDTA, 0.5 mM DTT, 1.15%KCl (w/v)) per milligram of tissue. A protease inhibitor cocktail (Sigma, St. Louis, Missouri) was added to the phosphate buffer immediately prior to use at a ratio of 1∶1,000. Samples were centrifuged at 600 *g* for 10 min at 4°C and the supernatant aliquoted for analyses. Protein concentrations of each sample were determined using the method described by Lowry et al [49].

Samples were loaded on 10% SDS-polyacrylamide gels and transferred to a PVDF membrane. Membranes were blocked with 5% BSA (wt/vol) in Tris-buffered saline with 0.1% Tween (vol/vol) (TBST) and incubated in primary antibody: RND3 (Abcam, Cambridge, MA; ab50316, 1∶1000); FOS (Abcam; ab16902, 1∶1000); total p38MAPK (Cell Signaling Technology, Danvers, MA; no. 9212, 1∶1000); p38MAPK (Thr180/Tyr182) (Cell Signaling Technology; no. 9215, 1∶1000); total GSK-3β (Cell Signaling Technology; no. 9315, 1∶1000); GSK-3β (Ser 9) (Cell Signaling Technology; no. 9323, 1∶1000), β-actin (BD Biosciences, Mississauga, ON; no. 612657, 1∶10000). After washing in TBST, membranes were incubated in either HRP-linked anti-rabbit or anti-mouse IgG secondary antibody (Amersham Biosciences, Piscataway, NJ; no. NA934V, 1∶6000), washed with TBST and developed using ECL (Amersham Biosciences; model no. RPN2106). Membranes were exposed to x-ray film (Biomax XAR; Kodak, Rochester, New York) which were then scanned with a Dell 920 scanner at 300 DPI and saved in TIF file format. Using Image J v1.40 g software (National Institutes of Health, Bethesda, Maryland), background noise was removed and bands in the region of interest were selected for analysis. Individual profile plots were generated and area under the curve measured in arbitrary units (AU).

### Statistical and bioinformatics analysis

Student's unpaired t-tests were used to determine differences in subject characteristics and total work. A 2-way repeated measures ANOVA (supplementation protocol × time) was used to assess differences in LDH, E2 concentration, testosterone concentration, protein levels and the linear 2^−δδCT^ data set for gene expression measured with RT-PCR using computerized software (Statistica, Statsoft). When statistical significance was achieved, Tukey's honestly significance difference post-hoc test was used to determine the significance among the means. STATISTICA for Windows 5.0 (Statsoft, Tulsa OK) was used to perform t-tests and ANOVAs. The threshold for significance was set at P≤0.05. Data are presented as mean ± SEM unless otherwise indicated.

Gene array data analyses were done comparing baseline, post supplementation, 3 and 48 hours post exercise using simple paired t-test on log_2_ expression ratios. Genes were ranked by p-value and the inference reported following adjustment for multiple testing using the FDR and the Benjamini and Hochberg method. Among those genes with an adjusted q-value (based on FDR) of <0.05, we used hierarchical clustering (based on the HOPACH algorithm) to find groups of genes with similar profiles across the subjects.

## Results

### Subject and work characteristics

CON and EXP groups were not different in age, weight, height, body fat percentage, average thigh cross-sectional area or total work completed (Table 2). All subjects completed the required 150 eccentric contractions.

**Table 2:** Subject and eccentric exercise trial characteristics.

	CON	EXP
No. of subjects	9	9
Age, yr	21.1±0.8	20.9±0.9
Height, cm	181.6±2.0	180.9±1.4
Weight, kg	73.4±3.8	80.4±4.6
Body fat, %	14.7±1.7	20.1±2.4
Quadriceps CSA, cm^2^	74.4±3.3	78.5±4.1
Work, kJ	24.9±2.9	25.1±1.3

Values are means ± SEM.

### E2 and testosterone concentration were affected by supplementation with E2

Following 8 days of supplementation serum E2 concentration increased by 146% (P<0.001) and testosterone concentration was reduced by 26% (P = 0.01) in the EXP group (Table 3). Both hormone concentrations remained unchanged in the CON group.

**Table 3:** Serum hormone concentrations after 8d of supplementation with either placebo (CON) or E2 (EXP).

Estradiol (pg/ml)	BL	PS
CON	36.4±2.5	38.4±2.8
EXP	38.8±4.0	95.4±11.7***

Testosterone (nmol/L)	BL	PS
CON		14.6±2.0
EXP		10.8±2.1**

Values are mean ± SEM.

** P<0.01.

*** P<0.001.

N = 9/group.

### Eccentric exercise induced muscle damage

The appearance of the muscle protein LDH in serum is an indirect indicator of muscle membrane damage. LDH activity was elevated 13.8% (P<0.05) 48 hours after exercise (Table 4). The EXP values did not differ from the CON values at any time.

**Table 4:** Serum lactate dehydrogenase activity following 150 eccentric contractions in CON and EXP groups.

	Baseline	3 hours	48 hours*
CON (U/L)	152.3±8.6	166.1±6.8	166.3±10.9
EXP (U/L)	140.7±9.7	158.1±9.2	165.4±16.3

Values are mean ± SEM.

*P<0.05 main effect for group compared to baseline.

CON (N = 7), EXP (N = 8).

### Microarray data identifies altered mRNA expression during recovery from eccentric exercise that is not affected by E2

Eccentric exercise significantly increased the early mRNA expression of 310 genes at 3H. DNA microarray analyses did not identify differential mRNA expression of any gene as a result of E2 at any time (we could not reject the global null that E2 was independent of mRNA expression of all genes represented on the chip). For this reason, microarray data at each timepoint was collapsed between groups, increasing the sample size to 18 subjects. Mean fold change for genes with ratios greater than 1 at 3H was 2.3±0.1. In addition, 301 genes had ratios less than 1 at 3H with a mean fold change of 0.8±0.01. By 48H, all genes had expression values that were not different from baseline. The complete data set is freely available at GEO (accession no. GSE19062).

Of the genes differentially expressed at 3H, we identified 25 that participate in two signaling cascades for muscle growth and adaptation: ras homologue gene family, member A (RHOA) and nuclear factor of activated T-cells (NFAT) (Table 5). Other regulators of muscle growth and remodeling not directly involved in RHOA or NFAT signaling that were also highly induced included: ATF3, MYC, XIRP1, HBEGF and DNAJB4 (Table 5).

**Table 5:** Fold change of gene expression after 3 hours of recovery from eccentric exercise using DNA microarray analysis (n = 18).

Categories and Gene Names	Accession Number	Fold change at 3H	Potential relevant function
**Calcineurin regulation**			
regulator of calcineurin 1 (RCAN1), transcript variant 3	NM_203418.1	5.6	Calcinuerin regulation
regulator of calcineurin 1 (RCAN1), transcript variant 2	NM_203417.1	3.9	Calcinuerin regulation
**NFAT**			
nuclear factor of activated T-cells, (NFATC1), transcript variant 1	NM_172390.1	1.3	calcineurin transcription/regulation
glycogen synthase kinase 3 beta (GSK3B)	NM_002093.2	1.3	calcineurin transcription/regulation
**MAPK regulation**			
**Activation**			
mitogen-activated protein kinase-activated protein kinase 2 (MAPKAPK2), transcript variant 2	NM_032960.2	1.2	MAPKKK cascade
adrenergic, beta-2-, receptor, surface (ADRB2)	NM_000024.3	2.5	activation of MAPK activity
mitogen-activated protein kinase 6 (MAPK6)	NM_002748.2	1.3	MAP kinase activity
mitogen-activated protein kinase kinase kinase 6 (MAP3K6)	NM_004672.3	1.4	protein serine/threonine kinase activity
mitogen-activated protein kinase kinase kinase 8 (MAP3K8)	NM_005204.2	11.1	protein serine/threonine kinase activity
**Inactivation**			
protein phosphatase 2 (formerly 2A), catalytic subunit, alpha isoform (PPP2CA)	NM_002715.2	1.5	inactivation of MAPK activity
dual specificity phosphatase 16 (DUSP16)	NM_030640.1	1.8	inactivation of MAPK activity
dual specificity phosphatase 8 (DUSP8)	NM_004420.1	1.4	inactivation of MAPK activity
**RHOA**			
striated muscle activator of Rho-dependant signaling (STARS)	NM_139166.2	10.1	positive regulation of Rho protein signal transduction
Rho family GTPase 3 (RND3)	NM_005168.3	5.4	small GTPase mediated signal transduction
Rho guanine nucleotide exchange factor (GEF) 7 (ARHGEF7), transcript variant 2	NM_145735.1	1.3	RHOA positive regulation
Rho guanine nucleotide exchange factor (GEF) 12 (ARHGEF12)	NM_015313.1	1.2	RHOA positive regulation
Rho GTPase activating protein 24 (ARHGAP24)	NM_031305.1	0.8	RHOA negative regulation
**Actin cytoskeleton and transcription factor activity**			
actinin, alpha 1 (ACTN1)	NM_001102.2	1.4	actin cytoskeleton
diaphanous homolog 1 (Drosophila) (DIAPH1)	NM_005219.2	1.9	actin cytoskeleton organization and biogenesis
coronin, actin binding protein, 1C (CORO1C)	NM_014325.2	1.5	actin binding/cytoskeleton
filamin B, beta (actin binding protein 278) (FLNB)	NM_001457.1	1.5	actin binding/cytoskeleton
actin, alpha 2, smooth muscle, aorta (ACTA2)	NM_001613.1	1.7	actin filament
jun D proto-oncogene (JUND)	NM_005354.2	1.9	activated transcription factor component
v-fos FBJ murine osteosarcoma viral oncogene homolog (FOS)	NM_005252.2	14.8	activated transcription factor component
FBJ murine osteosarcoma viral oncogene homolog B (FOSB)	NM_006732.1	3.7	activated transcription factor component
v-maf musculoaponeurotic fibrosarcoma oncogene homolog F (avian) (MAFF), transcript variant 1	NM_012323.2	2.6	regulation of transcription
**Other regulators of muscle growth and remodeling**			
activating transcription factor 3 (ATF3), transcript variant 3	NM_001030287.1	28.4	transcription factor activity
Xin actin-binding repeat containing 1 (XIRP1)	NM_194293.2	13.6	actin cytoskeleton organization
v-myc myelocytomatosis viral oncogene homolog (avian) (MYC)	NM_002467.3	11.0	transcription factor activity
heparin-binding EGF-like growth factor (HBEGF)	NM_001945.1	7.0	growth factor activity
DnaJ (Hsp40) homolog, subfamily B, member 4 (DNAJB4)	NM_007034.3	6.1	heat shock protein binding

IPA identified several biological functions related to muscle growth and remodeling identified by the number of differentially expressed genes included in the function and the calculation of a P-value using a Fisher's Exact Test with a threshold for significance set at P≤0.05. They included: cancer (P<0.05, 233 molecules), gene expression (P<0.05, 146 molecules), cell assembly and organization (P<0.05, 69 molecules), cell morphology (P<0.05, 59 molecules) and skeletal and muscular system development and function (P<0.05, 50 molecules). In the same manner, IPA also identified three canonical pathways related to gene expression and growth/proliferation: ILK signaling (P = 0.005, 16/186 molecules), RAN signaling (P = 0.005, 4/23 molecules) and PI3K/AKT signaling (P = 0.006, 12/136 molecules).

### RT-PCR analysis provides additional genes involved in actin dynamics and regulation of RhoA and NFAT signalling

**Figure 1:**
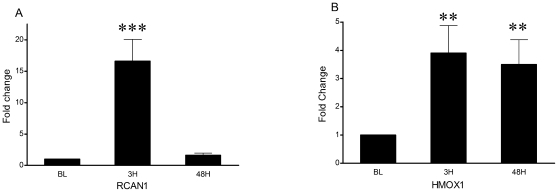
Expression fold changes in mRNA expression of genes in muscle from baseline after exercise protocol. Graph A – RCAN1 (N = 18). Graph B – HMOX1 (N = 18). 3H = 3 hours post exercise, 48H = 48 hours post exercise. Values are mean ± SEM. **Significant difference vs. baseline when collapsed across supplementation (P<0.01). ***Significant difference vs. baseline when collapsed across supplementation (P<0.001).

Targeted real time RT-PCR was conducted on several genes selected *a priori* for their involvement in recovery from skeletal muscle damage. Previous work using microarray analysis and RT-PCR identified the expression of novel genes following a similar eccentric protocol that are likely involved in the recovery and adaptation to damaging exercise [34]. Confirming the identification of two of its transcript variants in the microarray, RCAN1 mRNA content was highly elevated at 3H (16.6-fold, P<0.001) (Fig. 1A). Also induced was HMOX1 at both 3H (3.9-fold, P = 0.009) and 48H (3.5-fold, P = 0.002) (Fig. 1B).

**Figure 2:**
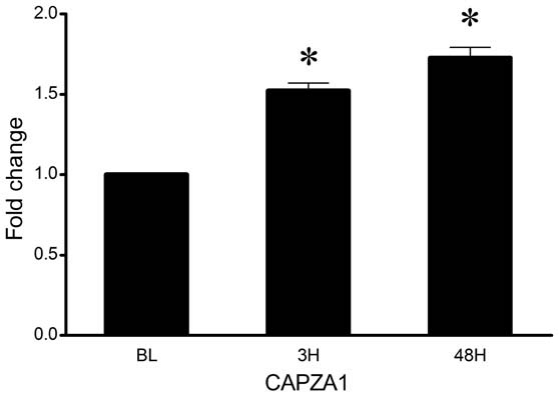
Expression fold changes in mRNA expression of CAPZA1 in muscle from baseline after exercise protocol. 3H = 3 hours post exercise, 48H = 48 hours post exercise. N = 18. Values are mean ± SEM. *Significant difference vs. baseline when collapsed across supplementation (P<0.05).

Expression of CAPZA1, a regulator of the growth of actin filaments, was increased at 48H (1.8-fold, P = 0.04) (Fig. 2).

### Signaling proteins and protein content are affected by eccentric exercise

**Figure 3:**
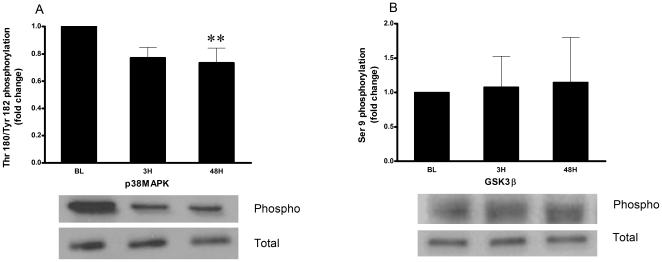
Fold change of phosphorylated/total ratio of signaling pathways from baseline after eccentric exercise. BL  =  baseline, 3H  =  3 hours post exercise, 48H = 48 hours post exercise. Graph A – p38MAPK (Thr ^180^/Tyr ^182^) (N = 18). Graph B – GSK-3β (Ser^9^) (N = 18). Values are mean ± SEM. **Significant difference vs. baseline when collapsed across supplementation (P<0.01).

Phosphorylated p38MAPK and GSK-3β negatively regulate NFAT by promoting its export from the nucleus. Phosphorylation (Thr^180^/Tyr^182^) of p38MAPK was significantly lower at 3H (0.77-fold, P = 0.07) and 48H (0.73-fold, P = 0.005) as a result of exercise with no effect of E2 (Fig. 3A). Phosphorylation (Ser^9^) of GSK-3β was not affected by either exercise or E2 (Fig. 3B).

**Figure 4:**
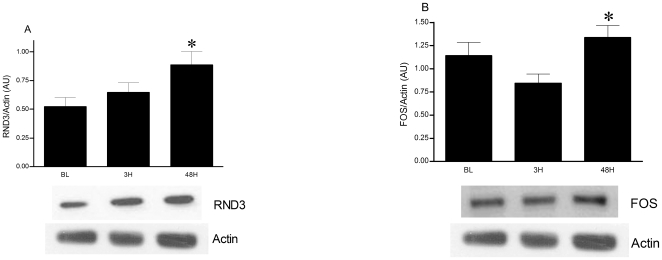
Western blot analysis of RND3 and FOS in skeletal muscle after eccentric exercise. BL  =  baseline, 3H = 3 hours post exercise, 48H = 48 hours post exercise. Graph A – RND3 (N = 18). Graph B – FOS (N = 14). Values are mean ± SEM. *Significant difference vs. baseline when collapsed across supplementation (P<0.05).

Two species highly expressed at 3H by the microarray were selected for western blot analysis. RND3 was significantly higher at both 3H (1.34-fold, P = 0.02) and 48H (1.39-fold, P<0.01) (Fig. 4A). FOS was significantly higher at 48 hours following exercise (1.16-fold, P<0.05) (Fig. 4B).

## Discussion

**Figure 5:**
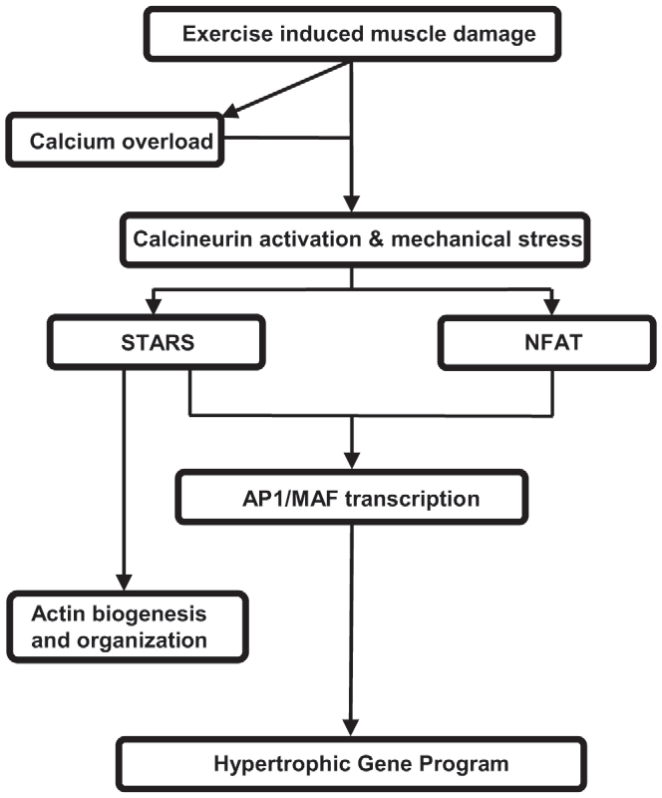
Schematic representation of the transcriptionally active pathways following exercise induced muscle damage. Eccentric exercise promoted greater expression of targets within the STARS/RhoA/AP1 and NFAT/AP1 signaling pathways for hypertrophy and actin biogenesis and organization.

The sex hormone E2 displays anti-oxidant and membrane stabilizing properties that could protect skeletal muscle from the effects of exercise induced muscle damage and influence genetic expression patterns [50], [51]. Using microarray, real time RT-PCR and protein analyses, we have identified that 8 days of E2 supplementation did not affect the myofibre transcriptome in men. However, a single bout of eccentric exercise did induce differential mRNA transcription in the hypertrophic signaling pathways RhoA and nuclear factor of activated T-cells (NFAT) (Fig. 5), changes in the phosphorylation status of related signaling proteins and the protein quantities of two of the upregulated genes.

**Figure 6:**
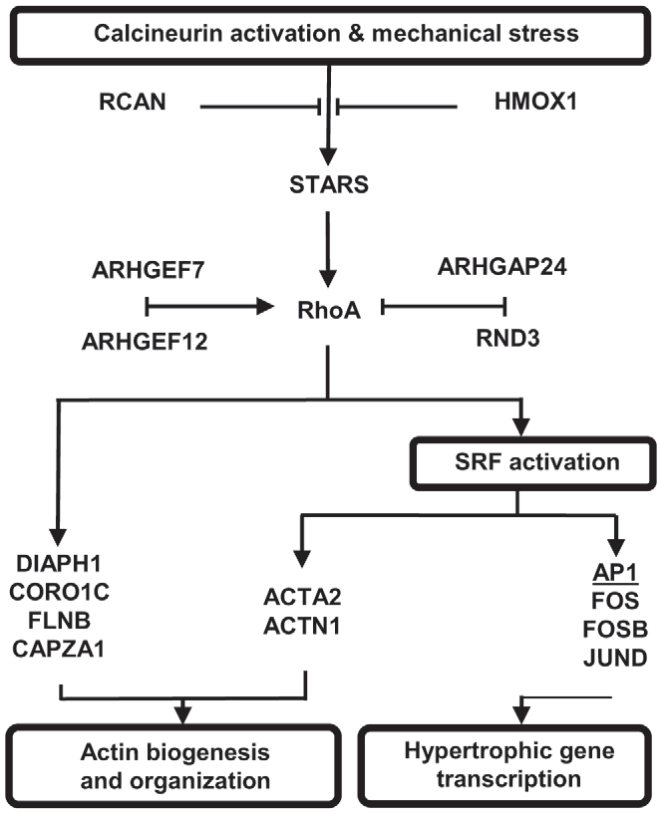
Regulatory and downstream targets of STARS transcriptionally active following a single bout of eccentric exercise. RCAN – regulator of calcineurin; HMOX1 – hemeoxygenase 1; ARHGEF7 and ARHGEF12 – Rho guanine nucleotide exchange factor 7 and 12; ARHGAP24 – Rho GTPase activating protein 24; RND3 – Rho family GTPase 3; DIAPH1 – diaphanous homologue 1; CORO1C – Coronin, actin binding protein 1; FLNB – Filamin B, beta; CAPZA1 – capping protein (actin filament) muscle Z-line alpha 1; ACTA2 – actin, alpha 2, smooth muscle, aorta; ACTN1 – actinin, alpha 1; AP1 – activator protein 1; FOS – FBJ murine osteosarcoma viral oncogene homologue; FOSB – FBJ murine osteosarcoma viral oncogene homologue B; JUND – Jun D proto-oncogene.

Of the genes affected early after exercise, one of the greatest inductions was observed in the novel actin binding protein striated muscle activator of Rho signaling (STARS). This protein is a muscle-specific transducer of cytoskeletal signaling in cardiac and skeletal muscle that responds to calcineurin activation and biomechanical stress [52], [53], [54], [55]. STARS stimulates growth through a mechanism requiring actin polymerization and Rho GTPase activation, increasing serum response factor (SRF)-mediated gene transcription (Fig. 6) [54], [55], [56], [57]. Originally identified in cardiac muscle, STARS mRNA content increases more than 3-fold following the hypertrophic signaling of pressure overload [52], [53]. More recently, Lamon *et al*. identified a 3.4-fold increase in STARS mRNA in human skeletal muscle following 8 weeks of resistance training [58]. Our measurement of more than a 10-fold increase suggests that this gene is very important for the early signaling for growth and remodeling following eccentric exercise.

A downstream target of STARS associated with skeletal muscle hypertrophy and adaptation is the Rho GTPase, RhoA [58], [59]. Rho GTPases are a family of small signaling G proteins that interact with effector proteins to regulate actin cytoskeleton, cell cycle progression and gene transcription [3], [60], [61], [62]. These molecular signals switch to an active GTP bound state under the control of Rho GEFs (guanidine exchange factors), and return to their inactive GDP bound state by Rho GAPs (GTPase-activating proteins) [60], [63], . For the first time, our array analysis has identified increased expression of two GEFs (ARHGEF7 and ARHGEF12) and reduced expression of a GAP (ARHGAP24) that specifically regulate RhoA [60], [65]. These gene expression modifications, if translated into altered protein quantities, could increase the potential for RhoA activation.

Although increased activity of RhoA protein is necessary for myogenesis induction, it must be downregulated before myotube formation can proceed [66], [67]. This is achieved by RND3, another negative regulator of RhoA activity whose upregulation is an essential step of myoblast fusion [62], [68], [69]. Our damaging exercise protocol resulted in an early induction of this gene at 3H and elevated protein content by 48H. Cell culture experiments have identified that in the presence of growth factors, RND3 mRNA increases of ∼1.7-fold result in greater protein content within 30 h [62]. This in turn inhibits RhoA activity and promotes myotube formation and elongation [62].

Once activated, RhoA signaling is associated with myogenesis and actin remodeling in various cell types through regulation of genes that include DIAPH1 [70], [71], CORO1C [72], FLNB [73] and CAPZA1 [74]. Through SRF activation, RhoA and STARS also mediate the induction of actin proteins ACTA2 [75], ACTN1 [76] and members of the AP1 transcription factor complex: FOS [76], [77], FOSB [77] and JUND [78]. Each of these genes was induced by eccentric exercise at 3H, as identified by our gene array and targeted real time RT-PCR analysis and our data indicate that the increased transcription of FOS was effectively translated into protein, increasing levels significantly by 48H. Although the number of studies investigating these genes after exercise is few, some support can be found for the upregulation of select downstream targets. Following eccentric exercise, the largest induction occurs with the transcription factor FOS 2–8 hours post exercise (23 to 38-fold increases) [10], [79]. Resistance training results in a 2.7-fold increase in ACTN1 after 8 weeks [58]. Thirty minutes of high intensity running increased the expression of FOS (7.0-fold) FOSB (17.8-fold) and JUND (7.6-fold) [80]. Given that the mRNA levels for STARS, associated regulatory and transcription factors and downstream targeted genes were all significantly elevated 3 h after exercise, it appears that STARS signaling through a RhoA/SRF pathway is important for early skeletal muscle remodeling following damaging exercise.

**Figure 7:**
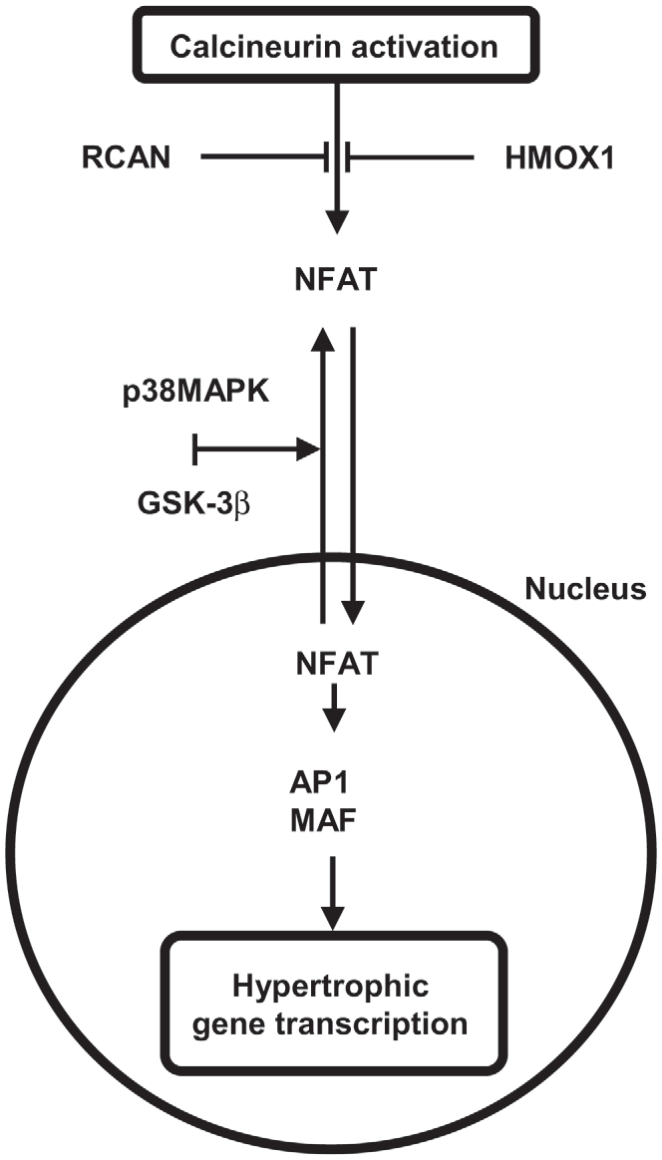
Regulatory and downstream targets of NFAT transcriptionally active following a single bout of eccentric exercise. p38MAPK – p38 mitogen activated protein kinase; GSK-3β – glycogen synthase kinase 3 beta; MAF – v-maf musculoaponeurotic fibrosarcoma oncogene homologue (avian).

A second calcineurin influenced signaling pathway identified in our microarray analysis to be transcriptionally active was nuclear factor of activated T-cells (NFAT). NFAT proteins exist in the cytoplasm of cells in a phosphorylated and inactive state [81]. The influx of calcium following sustained contraction or damage increases the binding of calcineurin to NFAT, dephosphorylating conserved serine residues and promoting translocation of NFAT into the nucleus [81], [82], [83], [84]. Once inside the nucleus, NFAT cooperatively binds to DNA with transcription factors AP1 and MAF initiating the transcription of prohypertrophic genes (Fig. 7) [85]. Our microarray analysis also identified a significant induction of the genes NFATc1 and MAF at 3H. Along with the greater expression of the AP1 complex components FOS, FOSB and JUND, an increased abundance of NFATc1 and MAF could improve signaling by NFAT.

The NFAT pathway interacts with mitogen activated protein kinases (MAPK) and glycogen synthase kinase-3β (GSK-3β) for coordination of the hypertrophic response [84], [86]. p38MAPK and GSK-3β act as a negative regulators of cardiac hypertrophy, rephosphorylating NFAT and promoting its export from the nucleus [83], [86], [87]. Although GSK-3β mRNA content was higher in the microarray, its activity did not change and would not have affected NFAT nuclear residence. GoMiner analysis identified MAPK regulation as a transcriptionally active biological process through the induction of four related kinases (MAPKAPK2, MAPK6, MAP3K6 and MAP3K8), three related phosphatases (PP2CA, DUSP8 and DUSP16) and one surface receptor (ADRB2). Western blotting confirmed that p38MAPK phosphorylation status was lower after exercise, reaching significance by 48H. Lower activity of p38MAPK would assist in the transcriptional activity of NFAT, and may also occur to inhibit the induction of apoptosis and necrosis [2].

It should be noted that molecules that function in the recovery and repair of skeletal muscle through other mechanisms were also induced by our exercise protocol. Similar to other reports, ATF3 [10], MYC [10], [34], XIRP1 [88], HBEGF [10] and DNAJB4 [10], [34], [35] were highly up-regulated (6.1 to 28.4-fold) early after exercise. In addition, the altered mRNA content of members of the signaling pathways ILK, RAN and PI3K/AKT identified them as being transcriptionally active. Their respective functions in actin cytoskeleton remodeling [89], transport across the nuclear envelope for gene expression [90] and protein synthesis [91] relate to the top biological functions returned by gene ontology analysis. Interestingly, the biological function that contained the greatest number of molecules was cancer (233 molecules), likely due to similar alterations to the regulation of cellular growth experienced with both exercise and cancer.

Maintained muscle contractions and exercise-induced damage to the sarcoplasmic reticulum and sarcolemma may result in the accumulation of excess calcium, known as Ca^2+^ overload [5], [92]. As a signaling molecule, Ca^2+^ binds to calcineurin, activating both STARS [52] and NFAT [81], [82], [83], [84], [93]. Unrestrained calcineurin activity can be regulated in skeletal and cardiac muscle by the inhibitors regulator of calcineurin 1 (RCAN1, aka MCIP1, DSCR1) [81], [94], [95], [96], [97] and hemeoxygenase 1 (HMOX1) [98]. Increased mRNA content of RCAN1 was identified in microarray and targeted real time RT-PCR, confirming a previous gene expression profile that also identified an increase following exercise (3.8-fold) [34]. RT-PCR also identified significant increases in HMOX1 at both time points after exercise, similar to the 8-11-fold induction following 5 days of resistance training [99]. Together, the upregulation of these two calcineurin inhibitors identifies the importance of regulating the elevated calcineurin activity that occurred following unaccustomed eccentric exercise [97].

The primary mechanism through which estrogens influence the growth, differentiation and function of tissues occurs via the estrogen receptors ERα and ERβ [31], [32], [100]. As transcription factors, active homodimers and heterodimers of ERα and ERβ bind to estrogen response elements (EREs) in nuclear and mitochondrial DNA, increasing the transcription of target genes [101]. In a nongenomic manner, E2 also interacts with a number of proteins that include endothelial nitric oxide synthase (eNOS) and MAPK, altering the signals that modulate cellular differentiation, migration and survival [102], [103], [104]. Our observation that the myofibre transcriptome was unaffected by E2 is surprising. Although women have higher E2 concentrations than men, protein quantities of ERα and ERβ are similar and our supplementation protocol successfully increased circulating E2 to the level seen during the luteal phase of the menstrual cycle in healthy women [105], [106]. This suggests that the differential expression between men and women of genes involved in metabolism and growth regulation [36], [37], [38] may result from factors beyond E2 alone; factors that may include variations in body composition, X-chromosome genes, and/or other sex hormones (i.e., progesterone).

These results indicate that E2 supplementation does not affect the transcriptional pattern in skeletal muscle following eccentric exercise in men. However, the stress of a single bout of exercise induced a transcriptional response in two signaling pathways, STARS/RhoA/AP1 and NFAT/AP1, providing important insights for future research into the early hypertrophic response.
